# Phloroglucinol Inhibits the Bioactivities of Endothelial Progenitor Cells and Suppresses Tumor Angiogenesis in LLC-Tumor-Bearing Mice

**DOI:** 10.1371/journal.pone.0033618

**Published:** 2012-04-09

**Authors:** Yi-Hong Kwon, Seok-Yun Jung, Jae-Won Kim, Sang-Hun Lee, Jun-Hee Lee, Boo-Yong Lee, Sang-Mo Kwon

**Affiliations:** 1 Laboratory for Vascular Medicine and Stem Cell Biology, Department of Physiology, School of Medicine, Medical Research Institute, Pusan National University, Gyeongsangnam-do, Republic of Korea; 2 Department of Biomedical Science, Laboratory for Functional Foods and Nutrigenomics, Department of Food Science and Biotechnology, CHA University, Kyunggi, Republic of Korea; Jawaharlal Nehru University, India

## Abstract

**Background:**

There is increasing evidence that phloroglucinol, a compound from *Ecklonia cava*, induces the apoptosis of cancer cells, eventually suppressing tumor angiogenesis.

**Methodology/Principal Findings:**

This is the first report on phloroglucinol's ability to potentially inhibit the functional bioactivities of endothelial progenitor cells (EPCs) and thereby attenuate tumor growth and angiogenesis in the Lewis lung carcinoma (LLC)-tumor-bearing mouse model. Although Phloroglucinol did not affect their cell toxicity, it specifically inhibited vascular endothelial growth factor (VEGF) dependent migration and capillary-like tube formation of EPCs. Our matrigel plug assay clearly indicated that orally injected phloroglucinol effectively disrupts VEGF-induced neovessel formation. Moreover, we demonstrated that when phloroglucinol is orally administered, it significantly inhibits tumor growth and angiogenesis as well as CD45^−^/CD34^+^ progenitor mobilization into peripheral blood *in vivo* in the LLC-tumor-bearing mouse model.

**Conclusions/Significance:**

These results suggest a novel role for phloroglucinol: Phloroglucinol might be a modulator of circulating EPC bioactivities, eventually suppressing tumorigenesis. Therefore, phloroglucinol might be a candidate compound for biosafe drugs that target tumor angiogenesis.

## Introduction

Phloroglucinol is a compound from *Ecklonia cava*, a species of brown alga. Recently, this biomolecule has attracted attention for drug synthesis because of its anti-inflammatory [1], anti-microbial, anti-allergic, and antioxidant activities [2], and human immunodeficiency virus (HIV)-1 reverse transcriptase and protease inhibitor activities [4]. However, little is known about its effect on progenitor cell-mediated tumor angiogenesis.

Tumor angiogenesis is a pivotal step in tumor growth because blood vessels generated by this pathophysiological process supply cancer cells with nutrients and oxygen, which are indispensable for the proliferation and survival of these cells. Emerging data shows that adequate inhibition of tumor angiogenesis can attenuate tumor growth [5]. Recently, the screening of natural products for anti-cancer properties has come into focus because these biomolecules cause few side-effects and have strong specific tumor cell targeting effects [6], [7], [8].

During tumor angiogenesis as well as vascular injury, circulating progenitor cells are dynamically mobilized from the bone marrow into the circulating blood, leading to vasculature remodeling [9], [10], [11], [12], [13]. Neovessel formation during tumor progression seems to be closely related to endothelial cells (EC)-mediated angiogenesis. Bone marrow (BM)-derived circulating progenitors including mesenchymal stem cells (MSCs), endothelial progenitor cells (EPCs) and hematopoietic precursor cells (HPCs) also contribute to this process [14], [15], [16]. However, therapeutic target molecules that effectively inhibit tumor angiogenesis by modulating stem/progenitor cells remain to be identified.

In the present study, we hypothesize that phloroglucinol is a good candidate anti-cancer biomolecule that modulates tumor growth during tumor progression by inhibiting the bioactivities of EPCs. Indeed, we demonstrated phloroglucinol's inhibitory effect on the cell migration, and capillary-like tube formation of EPCs using an *in vitro* functional assay. Importantly, orally treatment with phloroglucinol effectively disrupted vascular endothelial growth factor (VEGF)-induced *de novo* vessel formation in an *in vivo* murine matrigel plug assay. In the *in vivo* Lewis lung carcinoma (LLC)-tumor-bearing mice model, we showed that oral administration of phloroglucinol significantly inhibited tumor growth and angiogenesis as well as the mobilization of circulating EPCs, CD45^−^/CD34^+^ progenitor cells. This report highlights a novel role for phloroglucinol as a modulator of EPC bioactivities and suggest that it might be a potential cancer prevention drug.

## Results

### Characterization of EPCs from human umbilical cord blood (HUCB)

**Figure 1:**
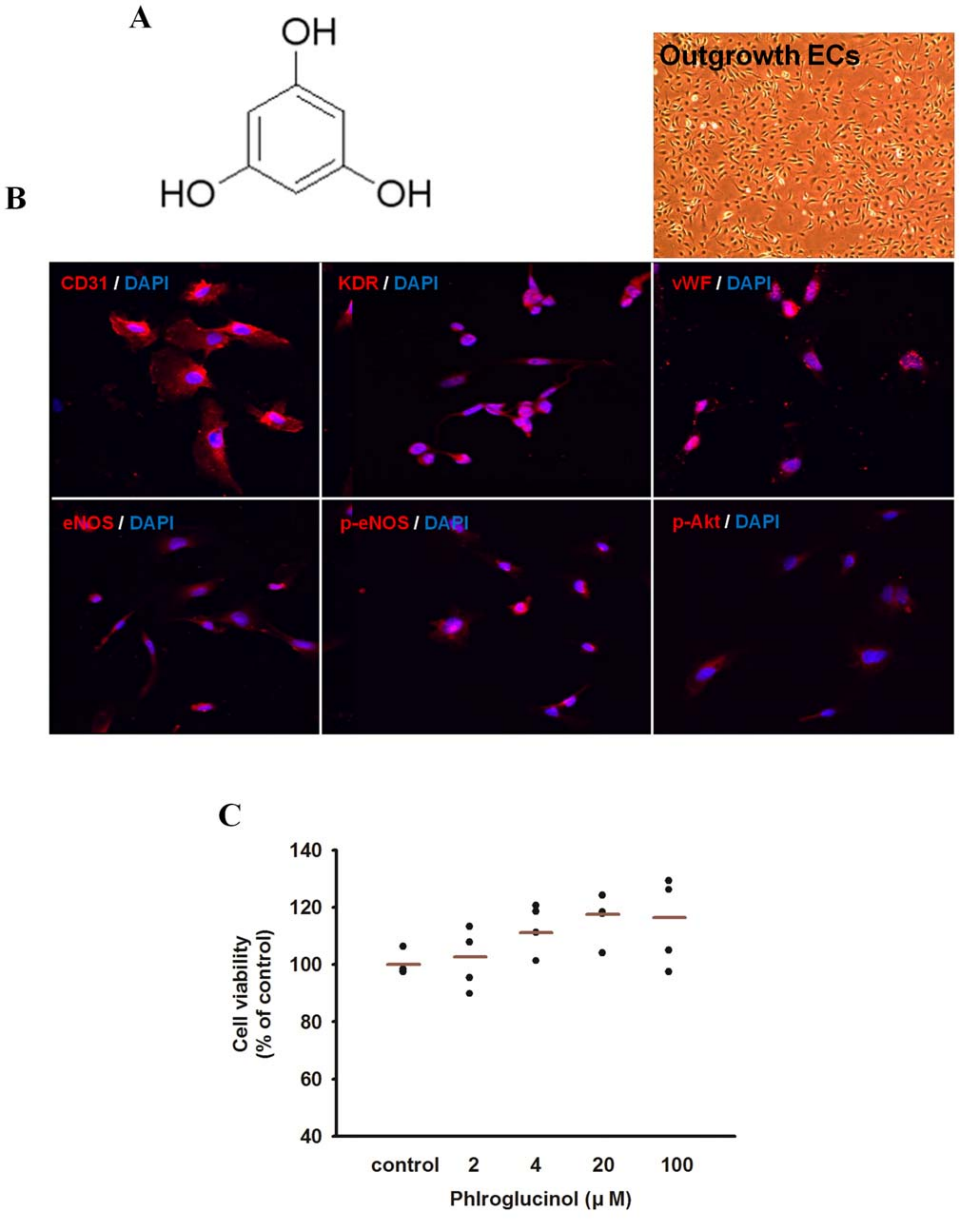
Effect of phloroglucinol derivatives isolated from *Ecklonia cava* on cell toxicity of EPCs. (A) Chemical structure of phloroglucinol: a class of natural products containing 1,3,5-trihydroxybenzene as the basic moiety. (B) Immunophenotyping of cell monolayers derived from HUCB EPCs by fluorescence microscope. A representative image is shown for HUCB EPCs. Immunophenotyping revealed that EPCs expressed endothelial cells lineage antigens including CD31, VEGFR-2 (KDR), VWF, eNOS, p-eNOS and p-Akt. (C) After treatment of phloroglucinol in EPCs, cell viability was examined using an MTT assay.

EPCs have been isolated from HUCB mononuclear cells (MNCs). Fluorescent staining was used to detect double positive cells binding of fluorescein isothiocyanate (FITC)-labeled Ulex europaeus agglutinin-1 (UEA-1) lectin and dioctadecyl-3,3,3′,3′-tetramethylindo carbocyanine (Dil)-labeled acetylated low density lipoprotein (data not shown). Immunophenotyping further revealed that *ex vivo* expanded EPCs expressed endothelial cells lineage surface antigens, CD31, VEGFR-2 (KDR), von Willebrand factor (vWF), eNOS, p-eNOS and p-Akt (Fig. 1B).

### Effect of phloroglucinol on cell toxicity of EPCs

In order to investigate cytotoxity of phloroglucinol in EPCs, cell viability assay was performend. As shown in Fig. 1C, Phloroglucinol did not reduce cell viability in EPCs at doses below 100 µM for 24 h. Therefore, concentrations of phloroglucinol ranging from 2 to 100 µM were selected for study on bioactivities of EPCs and tumor angiogenesis.

### Phloroglucinol inhibits the VEGF-induced migration of EPCs

**Figure 2:**
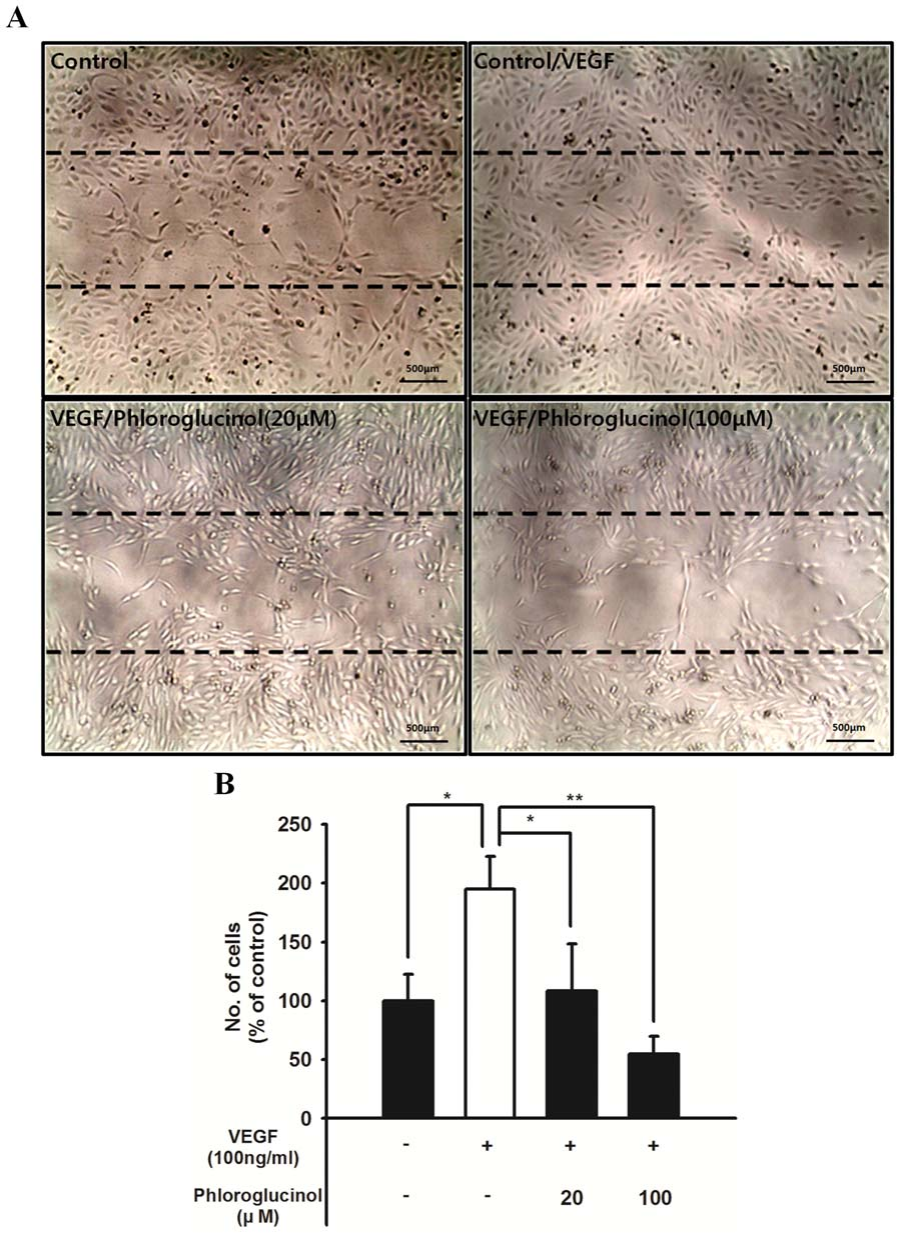
Effect of various concentrations of phloroglucinol on the migratory activity of EPCs in a wound healing assay. (A) *Ex vivo* cultured outgrowth ECs were subjected to a wound healing migration assay. Bar: 500 µm. EPCs were wounded and treated with 100 ng/ml of VEGF with or without 20 ìM or 100 ìM of phloroglucinol or a vehicle. (B) Bar graph represents the number of migrated cells. Fields were chosen randomly from various section levels to ensure objective sampling. In response to phloroglucinol, the VEGF-induced migratory activity of EPCs was significantly decreased.

Considering that BM mobilization kinetics of EPCs into peripheral blood (PB) is generally initiated by VEGF signaling, phloroglucinol may modulate the VEGF-induced migratory capability of EPCs. To test this idea, we next examined the effect of phloroglucinol on the migratory capability of EPCs using the wound healing assay. As shown in Figure 2A and 2B, induction of VEGF significantly repaired the wounded monolayer of EPCs. In contrast, phloroglucinol significantly reduced the VEGF-induced wounded area in a dose-dependent manner.

### Phloroglucinol inhibits the tube-forming capacity of EPCs

**Figure 3:**
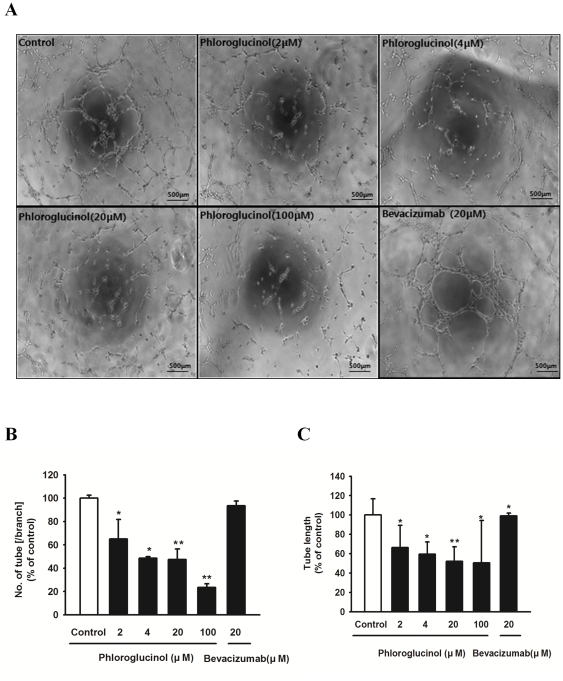
Effect of phloroglucinol on tubule-like structure formation of EPCs. (A) Representative tubular network structure of VEGF-stimulated outgrowths of ECs treated with or without phloroglucinol. Bevacizumab was used as a positive control. Bar: 500 µm. (B, C) Tube branches and total tube length were quantified using MacBiophotonics Images J software. Bar graph represents the number of intact loops in the capillary networks. Graph represents the length of tubes in the capillary networks (*P<0.05, **P<0.01).

We further identified the effect of phloroglucinol on the *de novo* capillary-like tubular formation of circulating progenitor cells (Fig. 3A). Treatment with phloroglucinol resulted in significant reduction in the number of branches and length of EPC tubes in a dose-dependent manner (Fig. 3B and 3C).

### Phloroglucinol suppresses tumor growth and tumor angiogenesis

**Figure 4:**
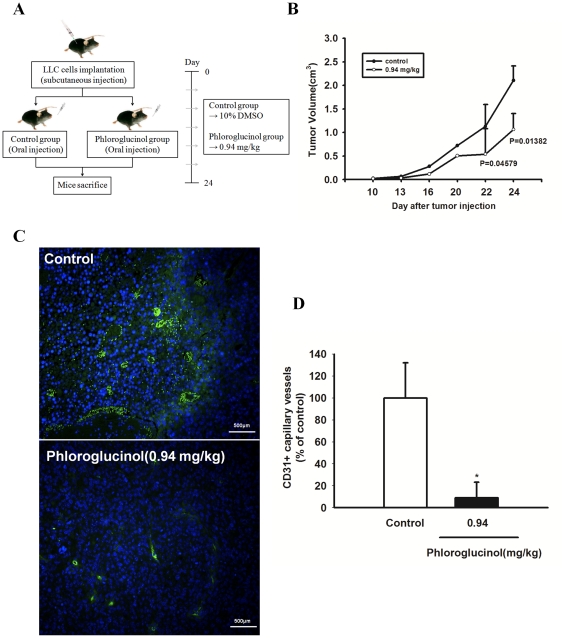
Effect of phloroglucinol on tumor growth and tumor angiogenesis in LLC tumor-bearing mice. (A) Male C57BL/6 mice were injected subcutaneously with 5×10^4^ LLC cells. LLC tumor-bearing mice were treated with DMSO solvent control and phloroglucinol at 0.94 mg/kg daily for 24 days after initiation of therapy. (B) Tumor growth was measured with calipers every 3 or 4 days using the formula V = height×length×depth (cm^3^). All data are represented as the mean tumor volume ± SE for the 7 animals in each group.. (C) Representative photomicrographs of CD31 capillaries in tumor sections stained with rat anti-mouse CD31 (green fluorescence), a typical endothelial marker. Nuclei were counterstained with DAPI (blue). Sections were photographed at ×100 magnification using an fluorescent microscope. Bar: 500 µm, (D) Quantification of the density of CD31^+^ capillary neovessels. The number of CD31-stained capillaries was counted using the Image J program. Fields were chosen randomly from various section levels to ensure objective sampling (*P<0.05).

In order to explore whether daily oral administration of phloroglucinol can suppress tumor growth and tumor-induced angiogenesis, we generated *in vivo* LLC tumor-bearing mice. To do this, we injected LLC tumor cells into male C57BL/6 mice, following which they were orally administered 0.94 mg/kg phloroglucinol (experimental group) or DMSO solvent (control group) daily for 24 days (Fig. 4A). At the time of death, all the mice treated with the vehicle only had a large tumor volume reaching 2.10±0.309 cm^3^. A significant decrease in swelled tumor mass (1.062±0.341 cm^3^) was observed when LLC cells (5×10^4^) were injected into a mouse flank together with phloroglucinol (Fig. 4B). To further determine the direct effects of phloroglucinol on tumor-induced angiogenesis, we analyzed the capillary density of the peritumoral region of each group by staining sections with CD31 antibodies. As shown in Figure 4C and 4D, treatment with phloroglucinol led to a significant reduction in the number of CD31^+^ capillary microvessels in the peritumoral region, suggesting that phloroglucinol might suppress tumor-induced angiogenesis *in vivo*. In order to investigate the direct role of phloroglucinol during EPC –mediated tumor angiogenesis, we analyzed the number of incorporated EPC in tumor vessel following orally administration of phloroglucinol. As shown in Figure S1, phloroglucinol significantly inhibited EPC incorporation into tumor-mediated vessels when isolated labelled EPCs applied into tail vein of tumor bearing mice, suggesting that phloroglucinol effectively modulated the contribution of EPC during tumor angiogenesis, partly regulating migratory capabilities of EPC.

### Effect of phloroglucinol on mobilization of circulating EPCs, CD45*^NEG^*CD34*^POS^*cells

**Figure 5:**
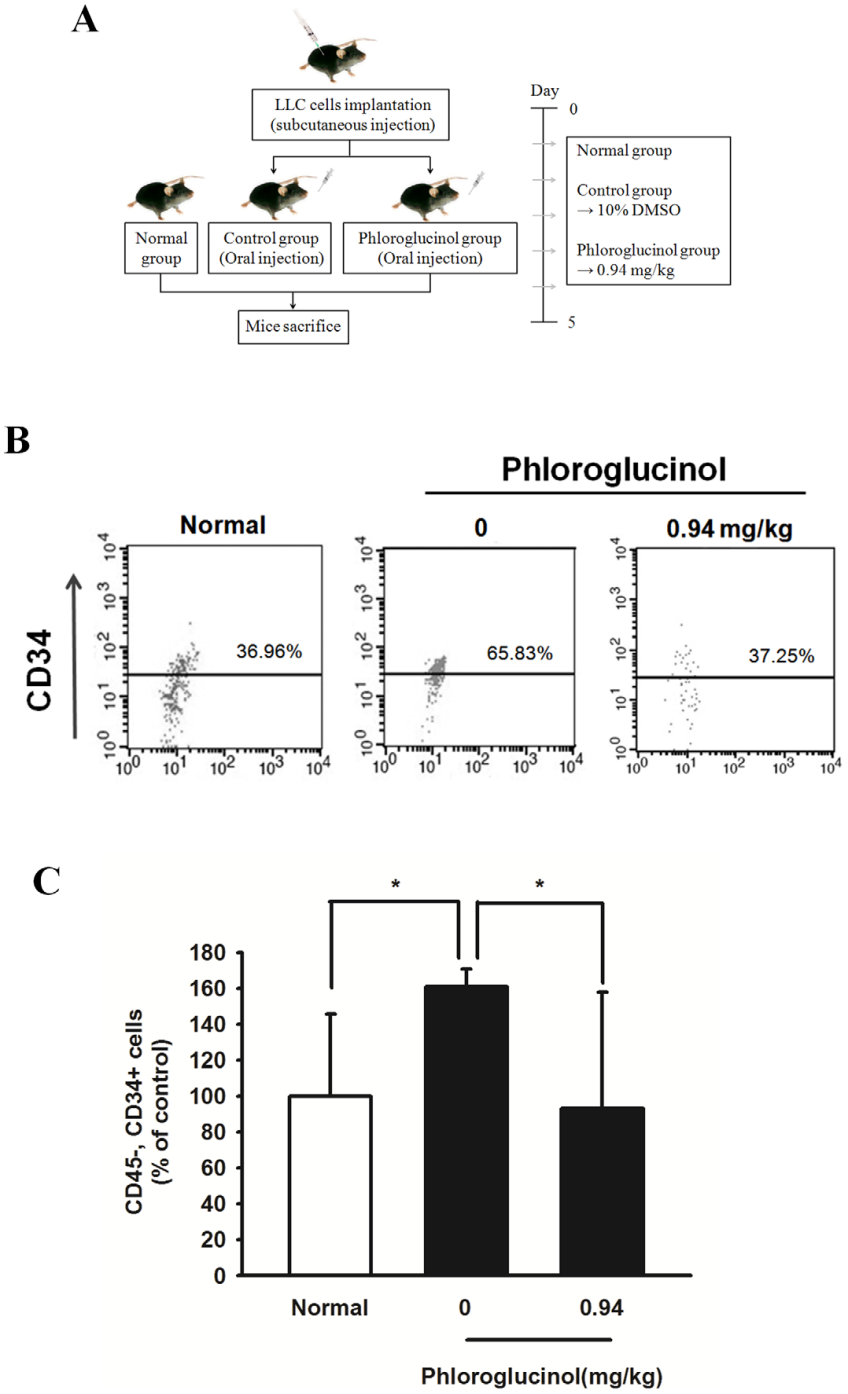
Effect of phloroglucinol on EPC mobilization in LLC tumor-bearing mice. (A) Schematic experimental schedule of *in vivo* EPC mobilization kinetics. After subcutaneous injection with 5×10^6^ LLC, the mice were orally administered DMSO vehicle (control) or 0.94 mg/kg phloroglucinol daily for 5 days after the initiation of therapy. Circulating MNCs were harvested at day 5 by Ficol-gradient centrifugation. (B) A subsequent gate was used to select the total CD45*^NEG^* cell population. Corresponding flow cytometric analysis was used to detect CD34*^POS^* cells in the gated CD45 *^NEG^* cell population. The EPC population was represented as CD45*^NEG^*/CD34*^POS^* cells. (C) Statistical difference between phloroglucinol after oral administration of both phloroglucinol and vehicle in LLC-tumor bearing mice. Bar graph represents marked differences in EPC frequency at day 5 after daily injection of phloroglucinol or vehicle (*P<0.05).

Since orally administered phloroglucinol in tumor-bearing mice exerts a significant inhibitory effect on the mobilization of circulating EPCs, we performed additional experiments to determine the effect of phloroglucinol treatment on the mobilization of EPCs from the bone marrow (BM) niche into circulating blood. Following subcutaneous transplantation (5×10^6^ cells) of LLC into the thighs of C57BL/6 mice, they were orally administrated 0.94 mg/kg phloroglucinol for 5 days (Fig. 5A). As shown in Figure 5B, the number of CD45*^NEG^*CD34*^POS^* cells, i.e., circulating EPCs, significantly increased compared to in normal mice. Importantly, oral administration of phloroglucinol for 5 days resulted in a significant reduction in the number of CD45*^NEG^*CD34*^POS^* EPCs circulating in PB.

### Phloroglucinol inhibits VEGF-induced *in vivo* angiogenesis

**Figure 6:**
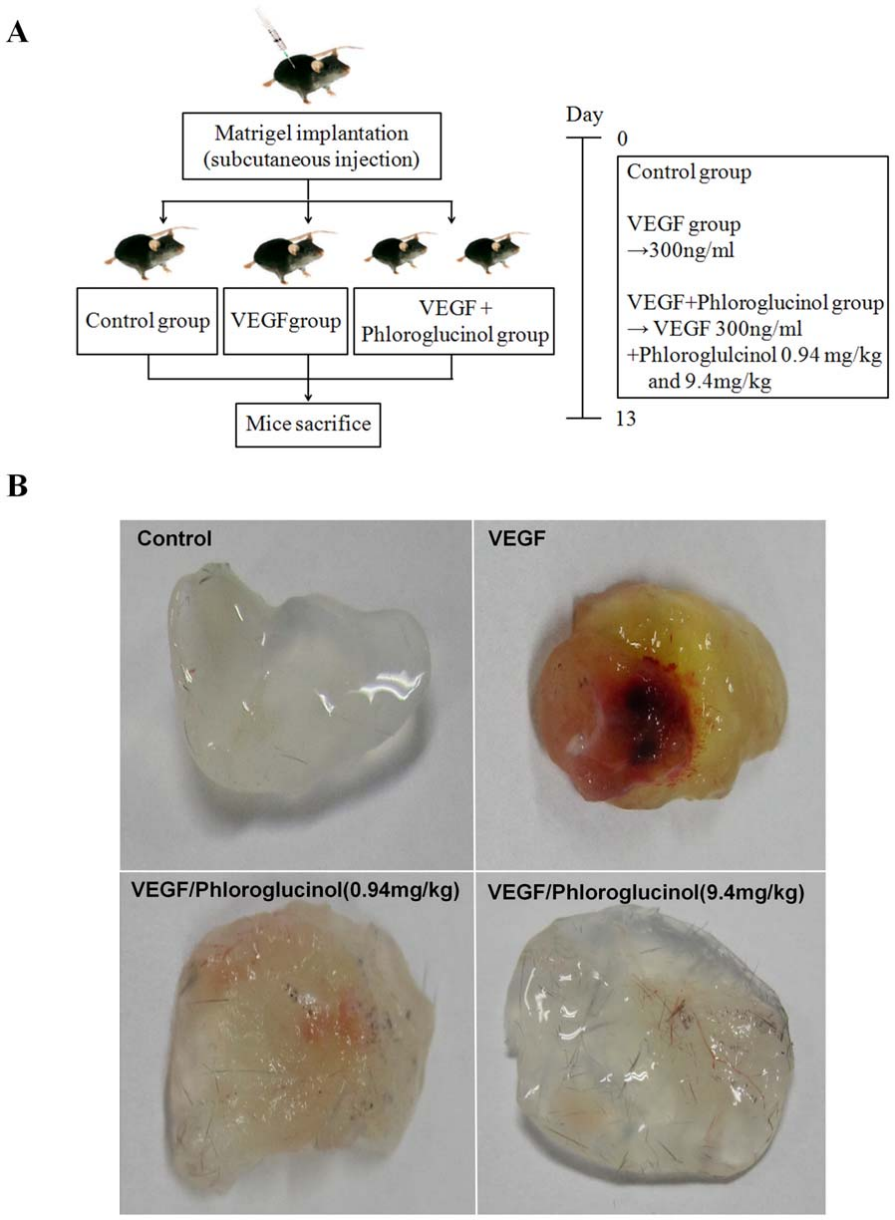
Phloroglucinol attenuated VEGF-dependent angiogenesis a in matrigel plugs assay. (A) Experimental protocols used in the VEGF-dependent matrigel plug assay. (B) Representative matrigel plug of each group at 13 days after injection. C57BL/6 mice (n = 5 per group) were subcutaneously injected with growth factor-reduce matrigel alone (none) or a combination of VEGF (300 ng/ml) and phloroglucinol (0.94 mg/kg and 9.4 mg/kg). VEGF loaded plugs from mice exhibited red color indicating abundant red blood cells.

**Figure 7:**
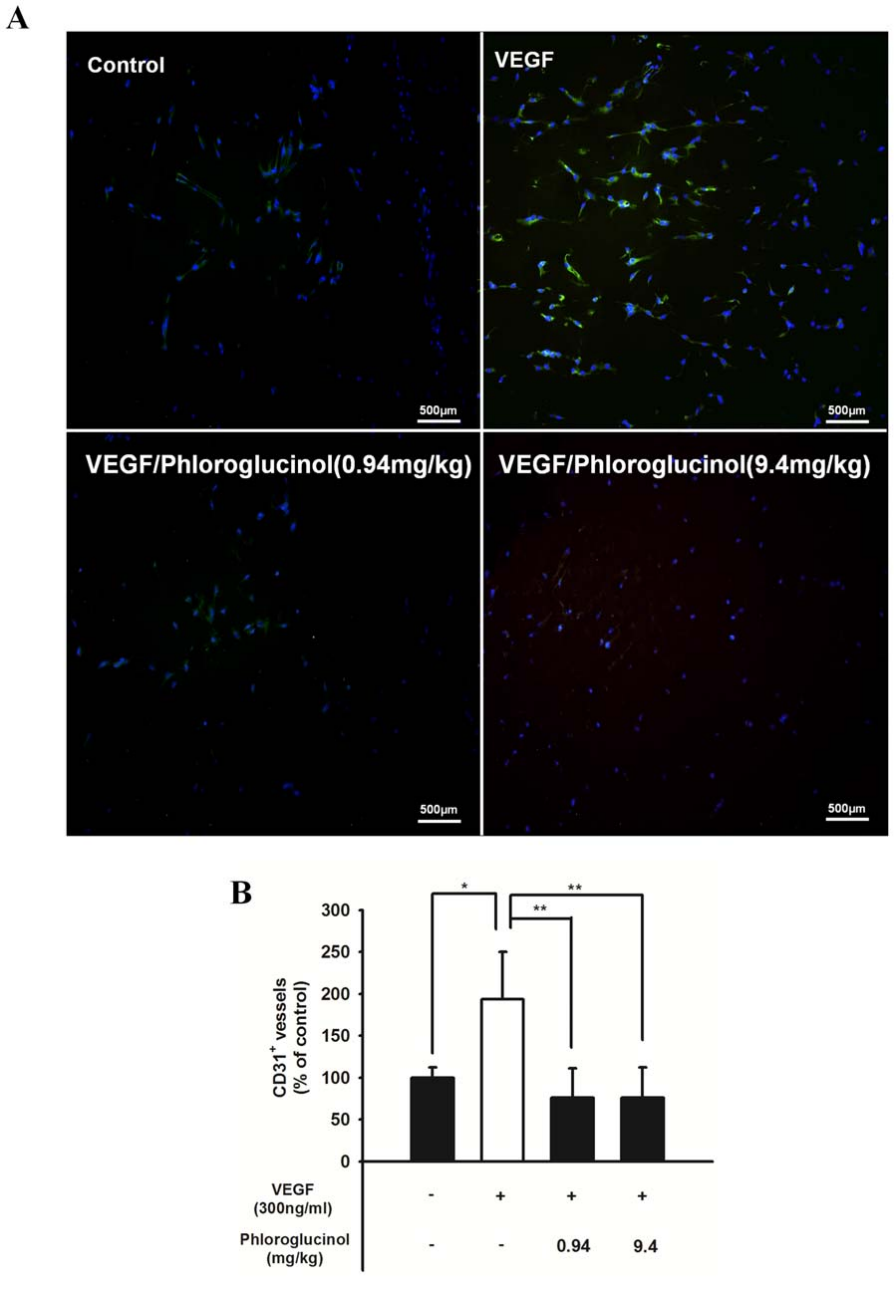
Phloroglucinol decreased the number of CD31(+) vessels a in matrigel plugs assay. (A) Representative photomicrographs of CD31-stained matrigel sections obtained from mice treated with the vehicle. After 13 days, the mice were sacrificed, and the matrigel plugs were removed. The histological sections were fixed with 4% paraformaldehyde and embedded in paraffin. Infiltrating endothelial cells in plugs stained during an immunohistochemistry assay of anti-CD31 antibody. Bar: 500 µm. (B) Quantitative assessment of CD31-positive capillaries (*P<0.05, **P<0.01).

In order to investigate the reason for phloroglucinol's anti-angiogenic activity in the *in vivo* angiogenesis model, we performed a matrigel plug assay (Fig. 6A). As shown in Figure 6B, the group with VEGF-loaded plugs yielded a red image, indicating an abundance of red blood cells in the newly formed vessels, while plugs with matrigel alone or with 0.94 mg/kg and 9.4 mg/kg phloroglucinol yielded light yellow images, indicating comparatively less blood vessel formation. These results suggest that phloroglucinol significantly reduces VEGF-dependent neovessel formation. To further examine the effect of phloroglucinol on capillary density, we performed immunohistochemical analysis by staining of CD31+ microvessels (Fig. 7A). As shown in Figure 7B, there was a significant decrease in the density of microvessels in the group with plugs with phloroglucinol plus VEGF as compared to the group with plugs with VEGF only.

## Discussion

Tumor-mediated neovascularization refers to the formation of new blood vessels from pre-existing vessels via the migration and proliferation of endothelial cells. This process requires multiple pathophysiological steps during the process of blood vessel formation, and it eventually contributes to tumor growth and angiogenesis [17], [18]. BM-derived EPCs are reported to play a significant role in tumor angiogenesis [19], [20]. Emerging evidence indicates impairments in the homing activities of BM-derived endothelial and hematopoietic precursor cells significantly blocks tumor angiogenesis and growth by decreasing the growth of early tumors. Blocking occurs because of interference in the angiogenic switch, which promotes tumor neovessel formation during tumor progression [21]. Importantly, the mobilization of EPCs from the bone marrow microenvironment to the peripheral blood may be an attractive target for anti-cancer treatments [9], [21], [22], [23]. This hypothesis explains why we were interested in understanding if the anti-tumor activities of phloroglucinol involve in inhibiting the bioactivities of EPCs and if these activities eventually suppress tumor growth and angiogenesis in the LLC-tumor-bearing mouse model.

Phloroglucinol compounds, both synthetic as well as natural, have a wide range of industrial applications because of their various biological activities such as anti-inflammatory, anti-microbial, anticancer, enzyme inhibitory, anti-allergic, anti-oxidant, and neurodegenerative activities [1], [2], [3], [24]. In general, hyperforin formulations, which are phloroglucinol derivatives, have been used to treat various types of cancer and precancerous tumors because they exert anti-proliferative bioactivities by activating the intrinsic apoptosis pathways of a panel of tumor cell lines, including carcinoma and leukemia cells as well as endothelial cells. Further, these formulations can be used to treat angioproliferative retinopathy and skin diseases, in which pathological angiogenesis occurs [25], [26], [27]. This study presents novel findings that oral administration of phloroglucinol significantly reduces *de novo* neovessel formation in LLC tumor tissue by affecting EPCs bioactivities including their migratory capability, tube-forming activity of EC outgrowths, and the mobilization of EPCs into the peripheral circulation. Among the proposed pivotal mediators of tumor-mediated neovascularization, VEGF signaling has been identified as the central signaling axis regulating proliferative activities, migration, and homing/incorporation of EPCs into sites of ischemic tissue [28], [29]. Thus, a wide range of preclinical strategies for the VEGF pathway has been shown to hinder tumor growth and angiogenesis [29], [30]. Increasing evidence shows that a VEGF-neutralizing antibody (bevacizumab) and VEGF receptor tyrosine kinase inhibitor (RTKIs), including sorafenib and sunitinib, are clinically approved anticancer treatments that target the VEGF/VEGF receptor axis [31], [32], [33]. Importantly, as shown in Figure S2, phloroglucinol more effectively inhibited tube forming capacity and migratotory capacity in low concentration as compared to those of bevacizumab. Based on the present findings, we concluded that phloroglucinol showed more superiority of phloroglucinol over well established, clinically proven antiangiogenic drug- bevacizumab, partly affecting *in vitro* bioactivities of EPCs in intracellular VEGF-mediate signal cascades, although bevacizumab can only modulate surface-mediated VEGF signaling.

This is the first report that phloroglucinol inhibits multiple VEGF-dependent EPC bioactivities, including the VEGF-dependent tube-forming capability and VEGF-dependent migratory capacity of EPCs *in vitro*. Further, this compound effectively abrogates VEGF-dependent neovessel formation *in vivo*, leading to delayed tumor progression and angiogenesis, partly through the modulation of EPCs *in vivo*.

Further studies are needed to investigate if the mechanical cascade underlying the phloroglucinol-dependent inhibition of tumor-mediated EPC mobilization during tumor angiogenesis can provide clues regarding cancer spread and metastatic growth since blockage of the process by phloroglucinol may be a significant target for therapeutic treatments [34], [35]. Interestingly, we recently observed that the effect of phloroglucinol on EPC mobilization seems to involve in altered VEGF dependent actin reorganization, not decreased VEGF levels in blood by phloroglucinol, suggesting that phloroglucinol might affect the bioactivities of EPCs including cytoskeletal changes (Figure S3). In particular, tumor cell-associated proteinases such as matrix metalloproteinases MMP2 and MMP9 [36], [37], [38] might play a role in the decline of mechanical barriers represented by extracellular matrices and may be closely related to the incorporation process and importance of EPCs during tumor angiogenesis.

Taken together, our data provide some evidence that phloroglucinol from edible and medicinal plants might be a potential drug for cancer prevention and/or chemotherapies.

## Materials and Methods

### Ethical statement

After obtaining informed consent, human umbilical cord blood was collected from healthy volunteers according to a protocol approved by the Ethics Review Board of the Hospital of the Pusan National University of YangSan, Korea. We have the list of the volunteers that university hospital can share. The Institutional Animal Care and Use Committee of the CHA University, Seoul, Korea approved all surgical interventions and post-operative animal care. The consent was written and approved. The approved protocol number is IACUC090017. Therefore, the Ethics Review Board approved this research including both the human and animal studies.

### Reagents

Phloroglucinol was purchased from Sigma-Aldrich (P1178). A solution of phloroglucinol was dissolved in dimethyl sulfoxide (DMSO, Amresco). The chemical structure of phloroglucinol is shown in Figure 1A.

### Cell culture of circulating EPCs and LLC cells

EPCs were cultured according to a previously described technique [39]. The Ethics Review Board of the Hospital of the Pusan National University of YangSan, Korea, approved the protocols, and the experimental study was conducted in accordance with the Declaration of Helsinki. In brief, mononuclear cells (MNCs) were isolated from human umbilical cord blood (HUCB). HUCB samples (approximately 60 ml each) were collected from fresh placentas with attached umbilical cords, using density gradient centrifugation with Ficol separating solution (Amersham). Freshly isolated MNCs were cultivated in 100 mm dishes coated with 1% gelatin (Sigma). The cultivation medium was endothelial basal medium (EBM)-2 (Lonza, Walkersville, MD) supplemented with 5% fetal bovine serum (FBS), human basic fibroblast growth factor (bFGF), human vascular endothelial growth factor (hVEGF), human insulin-like growth factor-1 (hIGF-1), human epidermal growth factor (hEGF), ascorbic acid, and GA-1000 (complete EGM-2 medium). After 4 days, non-adherent cells were removed, and the tightly attached fraction of cells were re-plated and cultured for another 3 days. Cultivation was continued by refreshing the EGM-2 medium until spindle-shaped colonies were formed after 14–21 days. Medium was changed daily for 7 days and then every other day until the first passage. Endothelial characteristics of the attached spindle-shaped EPCs were examined. Immunohistochemistry revealed that our late EPCs (*ex vivo* cultured outgrowth ECs) express several endothelial lineage markers using various antibodies against CD31, VEGF receptor-2 (KDR/Flk-1), von Willebrand factor (vWF), eNOS, p-eNOS and p-Akt. . Lewis lung carcinoma (LLC) cells were obtained from College of Pharmacy, Research Institute of Pharmaceutical Sciences, Kyungpook National University. The LLC cells were maintained in Dulbecco's Modified Eagle's Medium (DMEM, WelGENE) supplemented with 10% FBS and 1% penicillin/streptomycin (Gibco, Eggenstein, Germany) [40].

### MTT assay

The cytotoxic effects of the isolated compounds on cultured cells were measured using an MTT [3-(4,5-dimethylthiazol-2-yl)-2,5-diphenyltetrazolium bromide] assay. In brief, *ex vivo* expanded EPCs (1×10^4^ cells/well) were plated onto gelatin-coated, 96-well plates containing complete EGM-2 medium. After 24 h, the cells were serum starved in EBM-2 medium supplemented with 0.5% FBS for 12 h. They were then incubated with various concentrations of phloroglucinol in complete EGM-2 medium for 24 h. These were then incubated with 20 µℓ MTT solution (5 mg/ml, Amresco) for 4 h. Finally, 200 µℓ DMSO was added to solubilize the formed formazan, and the amount of formazan formed was determined by measuring the absorbance at 560 nm using a microplate reader. Viability of cells was quantified as a percentage compared to the control, and dose response curves were developed.

### Migration assay

*Ex vivo* expanded EPCs were cultured in 24-well plates at 1.5×10^5^ cells/well as confluent monolayers. The cells were incubated in EGM-2 medium for 24 h. Cells were starved in EBM-2 medium containing 1% FBS for 6 h and wounded. The wounded monolayer was then washed to remove cell debris, incubated for 14 h in 0.1% FBS in EBM-2 media containing various concentrations of phloroglucinol or bevacizumab and VEGF (100 ng/ml, Biobud). The area of the wound was recorded at the indicated time points using a light microscope. Migrated cells were analyzed under a light microscope.

### Tube formation assay

Matrigel (BD Biosciences, Bedford, MA) was dissolved at 4°C overnight, and 96-well plates were prepared with 55 µℓ matrigel in each well after coating and incubating at 37°C for 30 min. EPCs (2×10^4^) in 100 µℓ complete EGM-2 media were added with various concentrations of phloroglucinol or bevacizumab. After 6∼24 h of incubation at 37°C, EPCs' tube formation was assessed with a photomicroscope, following which each well was photographed at ×40 magnification under a light microscope. Tube branches and total tube length were calculated using MacBiophotonics Image J software.

### *In vivo* tumor growth

Male C57BL/6 mice were injected subcutaneously with 5×10^4^ LLC cells in DMEM. Drug treatment was initiated the day after tumor injection. LLC tumor-bearing mice (7 per group) were treated for 24 days with 150 µℓ of 0.94 mg/kg phloroglucinol or the solvent (10% DMSO). Tumor growth was measured with calipers every 3 or 4 days using the following formula: V = height×length×depth (cm^3^) [41]. We designed the *in vivo* tumor model of phloroglucinol to identify the possibilities as a tumor preventing drug, as phloroglucinol, a compound from *Ecklonia cava*, a species of brown alga and can be edible, biosafe natural bioproducts and can be used as a tumor preventing bio-drug. Therefore, we orally administrated phloroglucinol one day after tumor injection to evaluate the effect of phloroglucinol before initiating tumor.

### Contribution of EPCs during phloroglucinol-mediated tumor angiogenesis

To determine incorporation ratio of EPCs into tumor vessel by orally administrated phloroglucinol, the Dil-Ac-LDL-labeling-EPCs (1×10^6^ cells) were transplanted into tail vein of LLC-tumor-bearing C57BL/6 mouse (5 per group). Tumor tissue samples collected from the experimental animals were fixed 4% paraformaldehyde, rinsed in PBS, transferred to 30% sucrose in PBS at 4°C, and frozen in OCT compound. Frozen tissue sections were treated using goat anti-mouse CD31 antibody overnight at 4°C, followed by staining with FITC conjugated anti-goat antibody to identify capillaries in tumor.

### *In vivo* mobilization of circulating EPCs, CD45*^NEG^*CD34*^POS^*cells

Male C57BL/6 mice were injected subcutaneously with 5×10^6^ LLC cells in DMEM. Phloroglucinol treatment was initiated the day after tumor injection. LLC tumor-bearing mice (5 per group) were treated for 5 days with 0.94 mg/kg phloroglucinol. Control mice received equal amounts of 10% DMSO. Thereafter, mouse circulating EPCs were determined with flow cytometry using the following labels: CD45 (to exclude hematopoietic cell) (BD Pharmingen), hematopoietic/endothelial progenitor cell marker CD34 (BD Pharmingen) antibodies.

### Enzyme-Linked Immunosorbent Assay (ELISA)

Seven-week-old C57BL/6 mice were received subcutaneous injection with LLC cells (1×10^6^ cells). At starting day after tumor inoculation, they were orally administered 0.94 mg/kg phloroglucinol or DMSO solvent (control group) into LLC tumor bearing mice daily for 7 days. Using blood samples, ELISA was performed with a mouse specific VEGF ELISA kit (Quantikine M, R&D system, Minneapolis, Minn), as indicated in the manufacturer's protocol.

### Actin reorganization in response to VEGF

EPCs were seeded sparsely onto microscope glass coverslips placed within 24 well culture plates and allowed to grow for 1 day on change of the EGM-2 medium. Cells were starved in EBM-2 medium containing 0.1% FBS for 12 h. Incubated for 3 h in EBM-2 media containing various concentrations of phloroglucinol and VEGF (100 ng/ml, Biobud). Cells were fixed in 4% paraformaldehyde in PBS at room temperature for 20 minutes. Rhodamine-conjugated phalloidin was applied at room temperature for 60 minutes. After washing the coverslips in PBS, they were mounted onto object glasses using faramount mounting medium (Dako) and analyzed using confocal microscope (Olympus).

### Flow cytometry analysis

Mouse circulating EPCs were determined with flow cytometry using the following labels: CD45 (to exclude hematopoietic cell) (BD Pharmingen), hematopoietic/endothelial progenitor cell marker CD34 (BD Pharmingen) antibodies. Following red cell lysis, circulating EPCs were enumerated using flow cytometry (Becton Dickinson, Franklin Lakes, NJ), using gates to exclude dead cells, debris, and platelets. The percentage of stained cells was finally determined after comparing samples with matched isotype controls.

### Immunohistochemistry of CD31^+^ micro vessels

Vasculogenesis in the tumor tissue was determined by immunohistochemistry staining of CD31, a marker of endothelial cells. The immunohistochemical reactions were carried out on 5-µm-thick paraffin 4% paraformaldehyde-fixed-embedded sections from the phloroglucinol or the solvent treatment group, and paraffin sections of each tumor were stained for CD31 using a rat anti-mouse CD31 monoclonal antibody (BD Pharmingen). Specific binding of the primary antibody was visualized using Alexa488 coupled fluorescent secondary antibodies (Invitrogen). The fields were chosen randomly from various section levels to ensure the objectivity of sampling.

### *In vivo* matrigel plug assay

Growth factor-reduced matrigel (0.6 ml) containing 20 U of heparin and no VEGF, 300 ng/ml mouse VEGF, mouse VEGF plus 0.94 mg/kg and 9.4 mg/kg of phloroglucinol was injected subcutaneously into 7-week-old male mice. After 13 days, plugs were removed from the sacrificed mice. The matrigel plugs were fixed with 4% paraformaldehyde and embedded with paraffin. Next, 5-µm sections were stained in an immunohistochemistry assay with anti-CD31 antibody in order to visualize the endothelial cells. The fields were chosen randomly from various section levels to ensure the objectivity of sampling.

### Statistical analysis

Statistical comparison of 2 groups was performed using the Student's t-test. The results were analyzed using the Statview 5.0 software package (Abacus Concepts, Inc., CA). Scheffé's test was performed for multiple comparisons between each group after ANOVA. All data, which were obtained from at least 3 independent experiments, are expressed as means ± standard deviations.

## Supporting Information

Figure S1 **The direct role of phloroglucinol during EPC–mediated tumor angiogenesis.** (A) Representative photomicrographs of incorporated EPC (red fluorescence, Ac-LDL-labeled EPC) into CD31 (+) neovessel (green fluorescence). (B) Quantitative assessment of incorporated EPCs following orally administration of phloroglucinol. The bar graph represents a marked difference in the number of incorporated EPCs in phloroglucinol –treated mice.
(TIF)

Figure S2 **Direct comparision of** ***in vitro*** **ant-angiogenic effects between phloroglucinol and Bevacizumab.** (A) Effect of phloroglucinol and Bevacizumab on tubule-like structure formation of EPCs. Tube branches and total tube length were quantified using MacBiophotonics Images J software. Bar graph represents the number of intact loops in the capillary networks. Graph represents the length of tubes in the capillary networks (*P<0.05, **P<0.01). (B) Effect of phloroglucinol and Bevacizumab on the migratory activity of EPCs. EPCs were wounded and treated with 100 ìM of VEGF with or without 20 ng/ml or 100 ng/ml of phloroglucinol or Bevacizumab. (*P<0.05, **P<0.01). The bar graph represents a marked difference in the migratory activity of EPCs between phloroglucinol and bevacizumab.
(TIF)

Figure S3 **VEGF levels in blood of phloroglucinol-injected LLC tumor-bearing mice and cytoskeletal change of phloroglucinol-treated EPCs.** (A) VEGF levels were assessed by enzyme-linked immunosorbent assay in blood following orally administration of phloroglucinol in LLC tumor-bearing mice. (B) ltered actin reorganization in response to VEGF in phloroglucinol-treated EPCs. Rhodamine-conjugated phalloidin (red fluorescence) was applied at room temperature for 60 minutes and analyzed using confocal microscope. The representative image represents a marked difference in VEGF dependent actin reorganization in phloroglucinol-treated EPCs.
(TIF)
