# Supplementary figures and images for: Phloroglucinol Inhibits the Bioactivities of Endothelial Progenitor Cells and Suppresses Tumor Angiogenesis in LLC-Tumor-Bearing Mice
Source: PLoS One. 2012 Apr 9;7(4):e33618. doi: 10.1371/journal.pone.0033618 (PMC3322124; doi:10.1371/journal.pone.0033618)

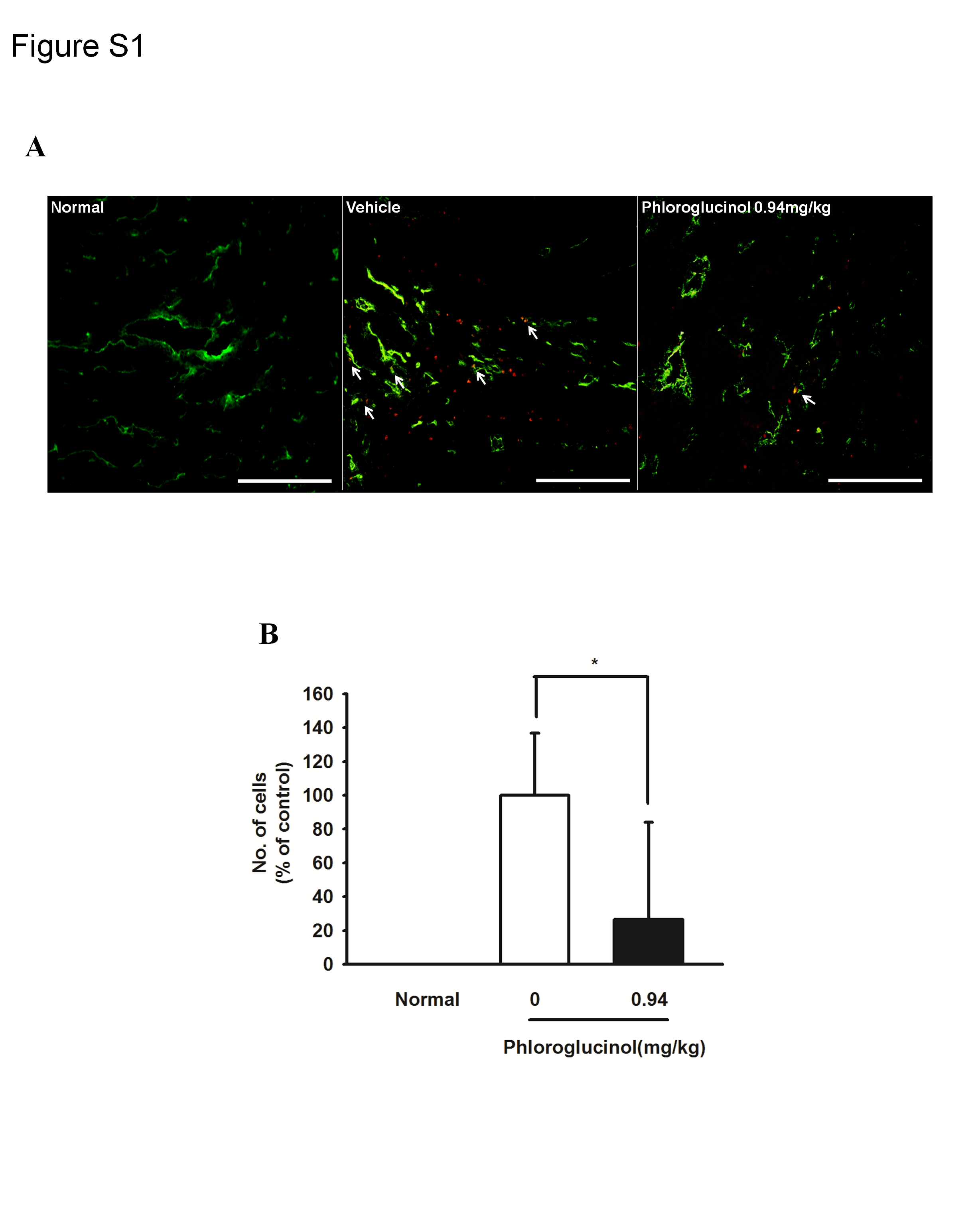

Supplement: Figure S1 — The direct role of phloroglucinol during EPC–mediated tumor angiogenesis. (A) Representative photomicrographs of incorporated EPC (red fluorescence, Ac-LDL-labeled EPC) into CD31 (+) neovessel (green fluorescence). (B) Quantitative assessment of incorporated EPCs following orally administration of phloroglucinol. The bar graph represents a marked difference in the number of incorporated EPCs in phloroglucinol –treated mice. (TIF) [file pone.0033618.s001.tif]

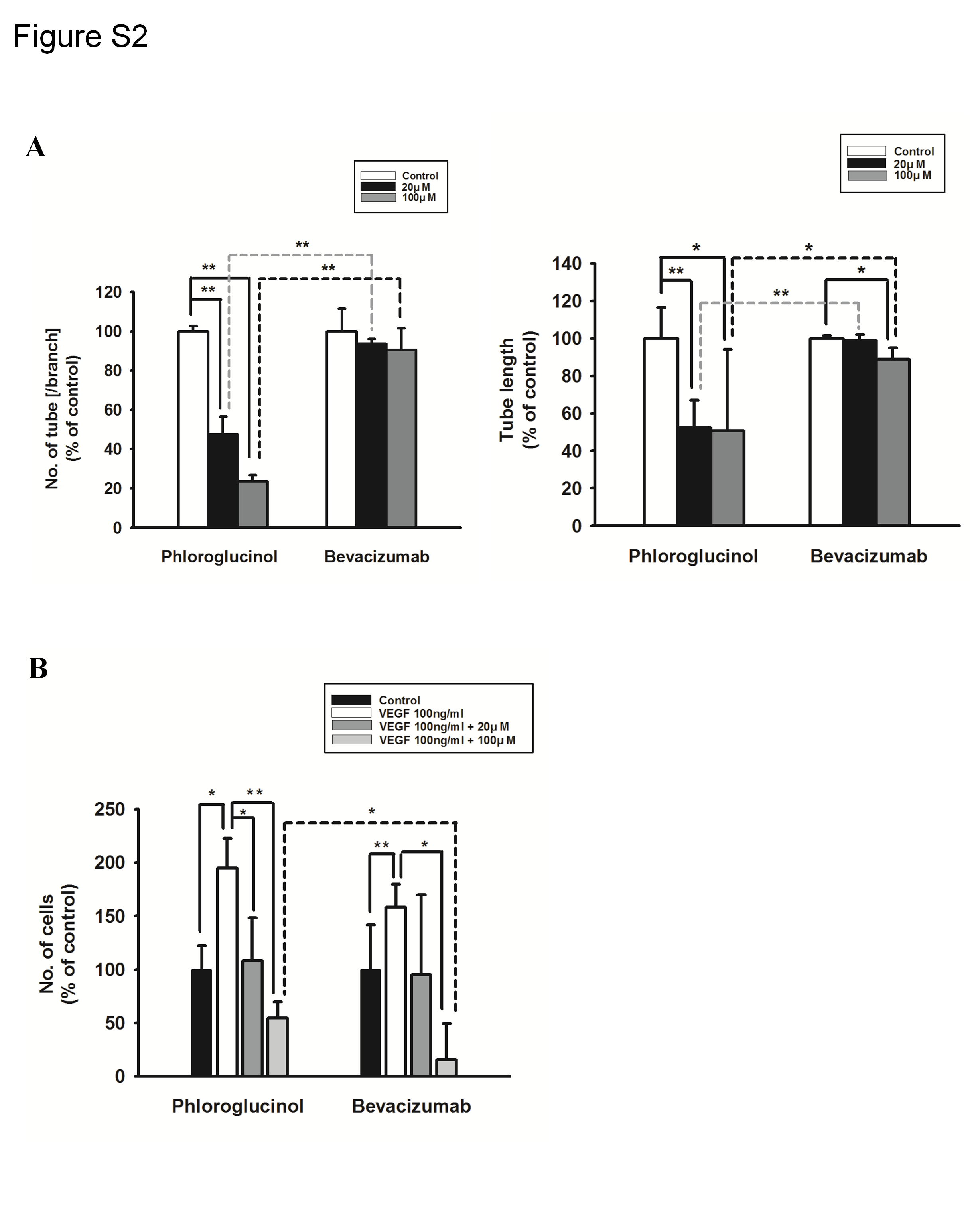

Supplement: Figure S2 — Direct comparision of in vitro ant-angiogenic effects between phloroglucinol and Bevacizumab. (A) Effect of phloroglucinol and Bevacizumab on tubule-like structure formation of EPCs. Tube branches and total tube length were quantified using MacBiophotonics Images J software. Bar graph represents the number of intact loops in the capillary networks. Graph represents the length of tubes in the capillary networks (*P<0.05, **P<0.01). (B) Effect of phloroglucinol and Bevacizumab on the migratory activity of EPCs. EPCs were wounded and treated with 100 ìM of VEGF with or without 20 ng/ml or 100 ng/ml of phloroglucinol or Bevacizumab. (*P<0.05, **P<0.01). The bar graph represents a marked difference in the migratory activity of EPCs between phloroglucinol and bevacizumab. (TIF) [file pone.0033618.s002.tif]

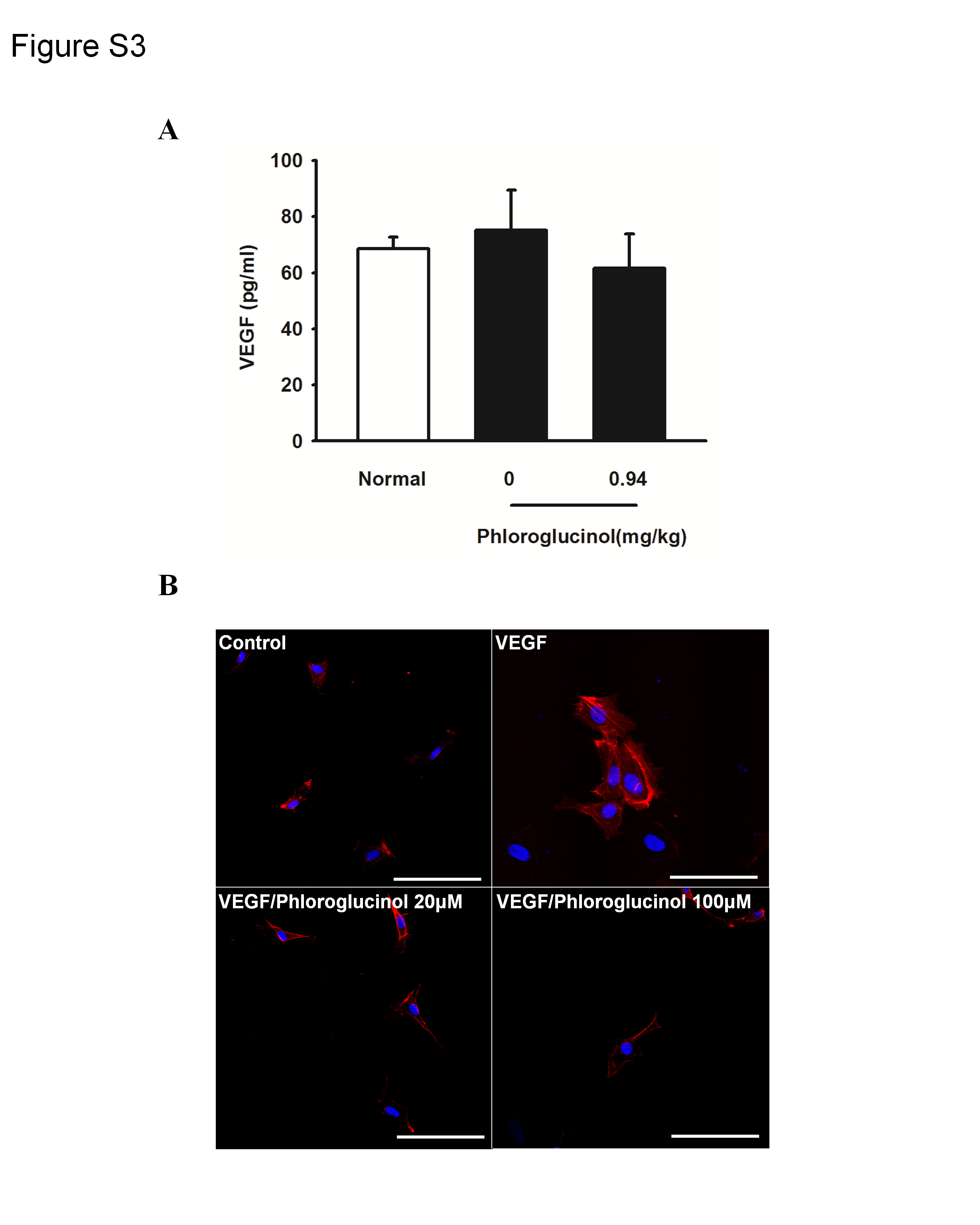

Supplement: Figure S3 — VEGF levels in blood of phloroglucinol-injected LLC tumor-bearing mice and cytoskeletal change of phloroglucinol-treated EPCs. (A) VEGF levels were assessed by enzyme-linked immunosorbent assay in blood following orally administration of phloroglucinol in LLC tumor-bearing mice. (B) ltered actin reorganization in response to VEGF in phloroglucinol-treated EPCs. Rhodamine-conjugated phalloidin (red fluorescence) was applied at room temperature for 60 minutes and analyzed using confocal microscope. The representative image represents a marked difference in VEGF dependent actin reorganization in phloroglucinol-treated EPCs. (TIF) [file pone.0033618.s003.tif]
